# Cochlear pathology in preeclamptic rats: protective effects of vitamin D and magnesium sulfate

**DOI:** 10.55730/1300-0144.5730

**Published:** 2023-10-12

**Authors:** Mustafa ALTINTAŞ, Enis HİDİŞOĞLU, Alexandra CERNOMORCENCO, Nuray ENSARİ, Didem Nevreste SONBAY YILMAZ, Özer Erdem GÜR, Hülya EYİGÖR, Züleyha Dilek GÜLMEZ, Erdoğan BULUT, Serap ŞIRVANCI, Selahattin KUMRU

**Affiliations:** 1 Department of Otorhinolaryngology, Antalya Training and Research Hospital, University of Health Science, Antalya, Turkiye; 2 Department of Drug Science and Technology, University of Turin, Turin, Italy; 3 Department of Histology and Embryology, Akdeniz University Faculty of Medicine, Antalya, Turkiye; 4 Department of Audiology, Faculty of Health Sciences, Istanbul University-Cerrahpasa, İstanbul, Turkiye; 5 Department of Audiology, Faculty of Health Sciences, Trakya University, Edirne, Turkiye; 6 Department of Histology and Embryology, Marmara University, İstanbul, Turkiye; 7 Department of Obstetrics and Gynecology, Faculty of Medicine, Akdeniz University, Antalya, Turkiye

**Keywords:** Preeclampsia, vitamin D, magnesium sulfate, embryonic weight, embryonic survival, cochlear degeneration

## Abstract

**Background/aim:**

This study investigated the possible degeneration in cochlear morphology induced by preeclampsia (PE) and the therapeutic/preventive effect of vitamin D (Vit D) and magnesium sulfate (MgSO_4_) used separately and together on feto-maternal outcomes.

**Materials and methods:**

We created PE in rats using a reduced uterine perfusion pressure (RUPP) animal model and recorded blood pressure (BP), embryonic survival (ES), and embryonic weight (EW) and evaluated cochlear morphology by electron microscopy.

**Results:**

The PE group had elevated BP, a decreased number and weight of live pups, and significant degeneration in the cochlea compared to the sham group. In the PEV group, we observed significant beneficial effects of Vit D supplementation at 14.5 and 19.5 dpc in terms of BP (p < 0.05), EW (p < 0.001), and cochlear degeneration compared to the PE group. In the PEM group, BP (p < 0.05) and cochlear degeneration nearly reached the level found in the sham group. However, although the EW was statistically different in the PE group, it did not reach sham group levels. We also observed that BP returned to sham level (p < 0.01) and noticed significant increases in the EW (p < 0.0001) and ES (p = 0.017) in the PEMV group compared to the PE group. According to the scanning electron microscope results, combined administration of VitD and MgSO_4_ is more effective than separate administration in improving cochlear degeneration induced by PE.

**Conclusion:**

The administration of Vit D and MgSO_4_ during pregnancy has beneficial effects on PE pathology and may play a significant role in preventing PE-related complications, including cochlear degeneration.

## 1. Introduction

Preeclampsia (PE) is a well-documented and pregnancy-induced hypertension syndrome accompanied by proteinuria typically occurring after the 20th week of pregnancy [1]. Although PE is one of the most important causes of maternal and perinatal morbidity and maternal death, the mechanisms responsible for its pathogenesis are still unknown. Since PE is responsible for the high percentage of maternal death during pregnancy, the design and development of preventive/therapeutic approaches are important for the early suppression and treatment of PE-related pathological conditions. Although 40% of PE-related deaths are associated with cerebrovascular complications, a multisystem disorder causes pathological outcomes and several dysfunctions in many organs, such as the brain, heart, eyes, and liver [2]. For example, significant increases in cerebral edema and blood-brain barrier permeability have been observed in a PE model induced by reduction of uterine perfusion pressure (RUPP) during pregnancy; in addition, the presence of white matter lesions in preeclamptic women has been reported [3–5]. Although the inner ear is also vulnerable to the same pathological conditions, such as ischemia, vasoconstriction, and autoimmune reactions, little is known about how it could be affected by PE. Recent studies have shown that PE is characterized as a state of oxidative stress resulting from an imbalance between reactive oxygen species (ROS) and antioxidant status, a significant factor in developing the disease [6,7]. One of the pathologic sources of ROS is the response to hypoxia/reperfusion and reduced organ blood flow followed by reperfusion. This condition leads to significant dysfunctions, specifically in end organs. Since the cochlea acts as an end organ, it can also be damaged by dysfunctions due to preeclampsia. Hearing problems observed in patients with PE strongly support this hypothesis. A study examining the possible damage of cochlea and sensorineural hearing loss in patients with PE showed that PE is a risk factor for otological pathologies and leads to cochlear damage and permanent hearing loss [2]. Another study evaluated outer hair cell function by distortion product otoacoustic emission in patients with PE. The findings indicate that PE has an adverse effect on the cochlear function by inducing the dysfunction of outer hair cells [8]. In addition, it was reported that patients with PE have a significantly higher hearing threshold than normal subjects [9]. Considering these studies’ findings, it can be suggested that PE has significant pathological outcomes in otology and nephrology, ophthalmology, cardiology, and neurology. Therefore, the current study aimed to investigate the preventive/therapeutic effects of magnesium sulfate (MgSO_4_), vitamin D (Vit D), and MgSO_4_ + Vit D administration on maternal and fetal outcomes, specifically cochlear damage in rats with PE obtained using a well-characterized method based on a RUPP rat model [10].

## 2. Materials and methods

### 2.1. Experimental design

All experimental protocols were conducted in accordance with the standards established by the Institutional Animal Care and Use Committee of Akdeniz University. We received ethical approval from Akdeniz University’s Local Committee on Animal Research Ethics (Ethics Approval Date and Number: 17.06.2019/92). The rats were obtained from Akdeniz University’s Animal Care Unit. Female Wistar rats aged 2–3 months weighing 250 to 300 g were housed appropriately and fed food and water ad libitium. The animals were kept in areas with a 12-h light-dark cycle and a temperature of 23 ± 1 °C. The female Wistar rats, bred in Akdeniz University’s Animal Care Unit Laboratory, were at a ratio of 4:1 with the male rats to facilitate mating. Pregnancy confirmation was performed by vaginal smears and checked in the early mornings. The day we observed the presence of spermatozoa was designated as 0.5 days postcoitum (dpc). At 14.5 dpc, 30 pregnant rats were divided into the following experimental groups:

Sham: sham-control pregnant rats were operated on in a similar way to that of RUPP mice but without ligation (n = 6).
PE: pregnant rats with PE receiving no treatment (n = 6).
PEM: pregnant rats with PE treated with MgSO_4_ (60mg/kg/day) between 14.5 dpc and 19.5 dpc by daily intraperitoneal injection (n = 6).
PEV: pregnant rats with PE treated with Vit D (300 IU) between 14.5 dpc and 19.5 dpc by daily gavage (n = 6).
PEMV: pregnant rats with PE treated with MgSO_4_ and Vit D (n = 6).

### 2.2. RUPP operation

At 14.5 dpc, we used a mixture of ketamine and xylazine for the anesthesia, and a midabdominal incision was made. This study used an established model of placental ischemia, the RUPP [10]. In this model, ligations are placed around pregnant rats’ ovarian and uterine vessels at 14.5 dpc. The complete ligation of the ovarian and uterine vessels has an adverse effect on and kills dams within a day or two after the operation, but tail-cuff blood pressure (BP) is not informative. Therefore, we preferred to tie the uterine arteries in the pregnant rats with a nylon thread of 0.2 mm in diameter, followed by removing the thread to provide a small space. After ligation, we sutured the abdominal incision, and the rats were given 4 mL/kg of a mixture containing 0.3% NaCl and 4% sucrose for rehydration.

### 2.3. Blood pressure (BP) measurement

Systolic BP was measured in all experimental groups at days 14.5 and 19.5 of pregnancy with the noninvasive BP system using tail-cuff plethysmography (Letica LE 5100, Panlab, Barcelona, Spain).

### 2.4. Tissue collection

An overdose injection of urethane (100 mg/kg) was used to sacrifice the animals, and a midline laparotomy incision was conducted to reach the uterine horns. The developed fetuses were counted and removed. After delivery of the pups, their weight and condition (dead or alive) were recorded. The cochlea tissues were isolated from each rat, washed in cold saline, and immediately collected in a 2% glutaraldehyde solution for ultrastructural examination.

### 2.5. Scanning electron microscopy (SEM)

The surface topography of the Corti organ was examined and photographed with a scanning electron microscope (SEM). Relevant changes (site of degeneration) were evaluated as normal inner hair cells (IHCs) and outer hair cells (OHCs) with intact V- or W-shaped stereocilia bundles and abnormal IHCs and OHCs with damaged stereocilia or loss of the normal V- or W-shaped stereocilia. Total absence of stereocilia and rupture of the cuticular plate were considered absent IHC and OHCs.

Subsequently, 2.5% glutaraldehyde was used to fix the tympanic cavity, and we dissected the tympanic bulla to reach the cochlear structure. After dissection, the cochlear structure was immersed in a phosphate buffer solution (pH = 7.3) for 12 h before decalcification in a 0.1 M Na-EDTA (Sigma-Germany) solution at room temperature for 2 weeks. After decalcification, the otic capsule was dissected asymmetrically from base to apex under a microscope (Olympus 1 × 71 S8-F3, Japan) to examine cochlear structures and regularly followed up with SEM for 3 days at + 4 °C PBS. Carbon monoxide was employed to dry the tissues at the critical drying point (CPD 010, Balzer Union, Liechtenstein) with carbon holders set on brass blocks. Under argon gas, the surface of the Corti organ was coated with gold (Bio-Rad SC502, VG Microtech, England). Routine follow-up procedures and SEM imaging (Zeiss EVO LS10, Germany) were then carried out.

### 2.6. Statistical analysis

All statistical analyses of the obtained data were performed using Windows SPSS 18.0 (IBM Corp., Chicago, IL, USA) software. The differences in BP and ES among the experimental groups were analyzed via Kruskal–Wallis one-way ANOVA on ranks, and all pairwise multiple comparisons were performed with the Mann–Whitney U test. A one-way ANOVA was used to determine the statistical significance of EW between the groups. Posthoc comparisons were carried out using Tukey’s test. Correlation values were obtained after performing Pearson’s correlation analysis and linear regression. The outcomes are shown as mean ± standard deviation. The significance threshold was set at p < 0.05. All experimenters were blind to animal experimental group membership during data collection and processing.

## 3. Results

### 3.1. Blood pressure changes

**Figure 1:**
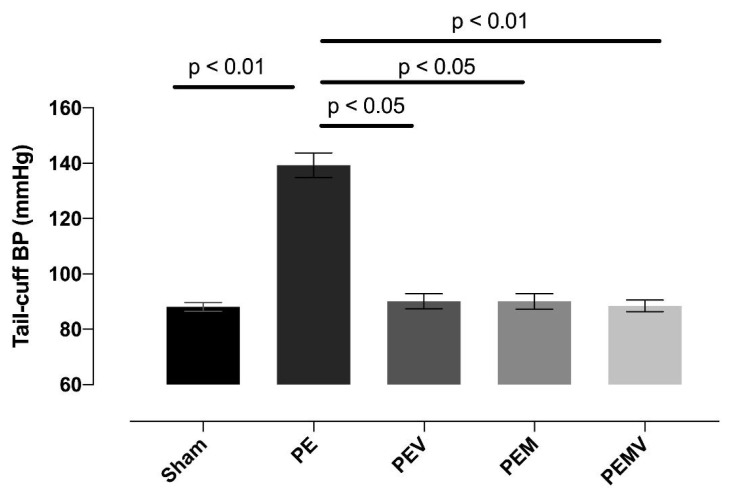
Tail-cuff blood pressure measurements at 19.5 dpc. Vit D and/or MgSO4 administration at 14.5 and 19.5 dpc has beneficial effects on hypertension induced by the RUPP operation.

The initial levels of systolic BP at 14.5 dpc were not different between the experimental groups, but the PE group showed statistically significantly higher BP than the sham group at 19.5 dpc (p < 0.01). Vit D supplementation significantly decreased the BP of the PE + Vit D (PEV) group compared to the PE group (p < 0.05). Like Vit D, the MgSO_4_ also significantly decreased the BP of the PE + MgSO_4_ (PEM) group compared to the PE group (p < .05). Moreover, Vit D + MgSO_4_ treatment led to a statistically significant decrease in the BP of the PE + Vit D + MgSO_4_ (PEMV) group compared to the PE group (p < 0.001), but there was no significant difference in the sham, PEV, PEM, and PEMV groups. The values of BP for the experimental groups (mean ± SEM) are given in Figure 1.

### 3.2. Fetal outcomes

#### Embryonic weight and survival

**Figure 2:**
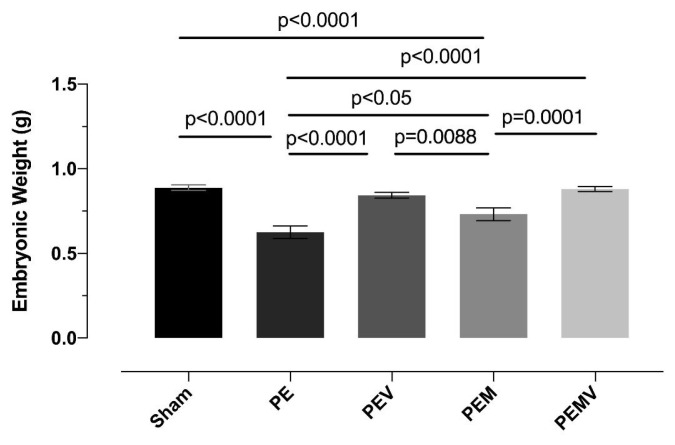
Embryonic growth restriction induced by the RUPP operation and the effects of Vit D and MgSO_4_ administration. Significant improvements in embryonic weight were observed after administration of Vit D and MgSO_4_ together and separately. However, the administration of MgSO_4_ alone is insufficient in restoring embryonic weight to the sham level.

As shown in Figure 2, at 19.5 dpc, the EW of the PE group was statistically lower than that of the sham group (p < 0.001). The embryos of the Vit D-treated PEV group had a final EW significantly higher than the PE group’s EW. (p < 0.001). Unlike the PEV group, the EWs of the PEM group treated with MgSO_4_ were slightly higher than those of the PE group (p < 0.05); in addition, EW in this group remained significantly lower than the sham group’s EW. (p < 0.001). In the PEMV group treated with Vit D and MgSO4, the EWs were significantly higher than the PE group (p < 0.001). We also observed significant differences between the PEV, PEM, and PEVM groups. The EWs of the PEV and PEMV groups were significantly higher than those of the PEM group (p = 0.088 and p = 0.001, respectively). However, there was no significant difference between the PEV and PEMV groups. The EW values for the experimental groups (mean ± SEM) are given in Figure 2.

**Figure 3:**
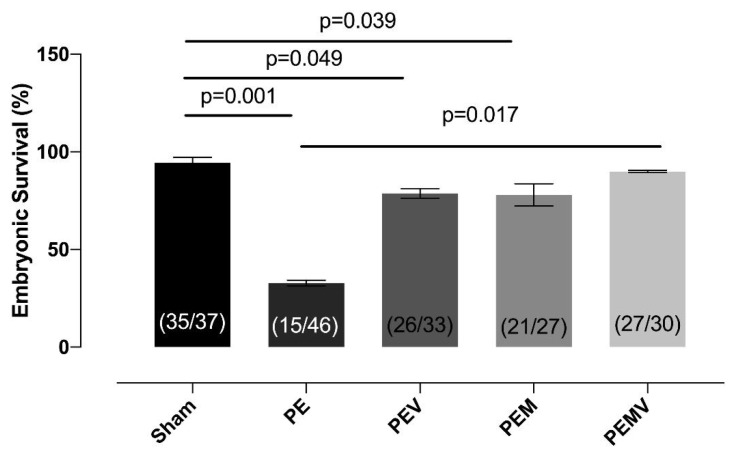
Decreased percentage of embryonic survival caused by the RUPP operation and possible protective effects of Vit D and MgSO_4_ administration. The administration of Vit D and MgSO_4_ together has more potent effects than administration of the two separately in terms of embryonic survival.

When we evaluated ES in the experimental groups at 19.5 dpc, we observed statistically significant differences. Multiple comparisons showed that the ES in the PE group was statistically lower than in the sham group (p < 0.001). Interestingly, Vit D and MgSO4 treatments led to increases in ES, but these increments did not reach statistical significance levels and remained lower in the sham group (sham vs. PEV group: p = 0.049; sham vs. PEM group: p = 0.039). However, the ES of the PEMV group treated with Vit D and MgSO_4_ was significantly higher than in the PE group (p = 0.017). The ES values for the experimental groups (mean ± SEM) are given in Figure 3.

##### Correlation analysis

We also performed Pearson’s correlation analysis to evaluate whether a significant relationship between BP and fetal outcomes existed, revealing a significant negative correlation between systolic BP and ES and ES at 19.5 dpc (Pearson’s r =−0.913; p < 0.001 and Pearson’s r =−0.727; p < 0.001, respectively).

### 3.3. Evaluation of cochlea by SEM

In this study, we evaluated stereocilia degeneration in the surface anatomy of the Corti organ for loss of HCs in all groups.

**Figure 4:**
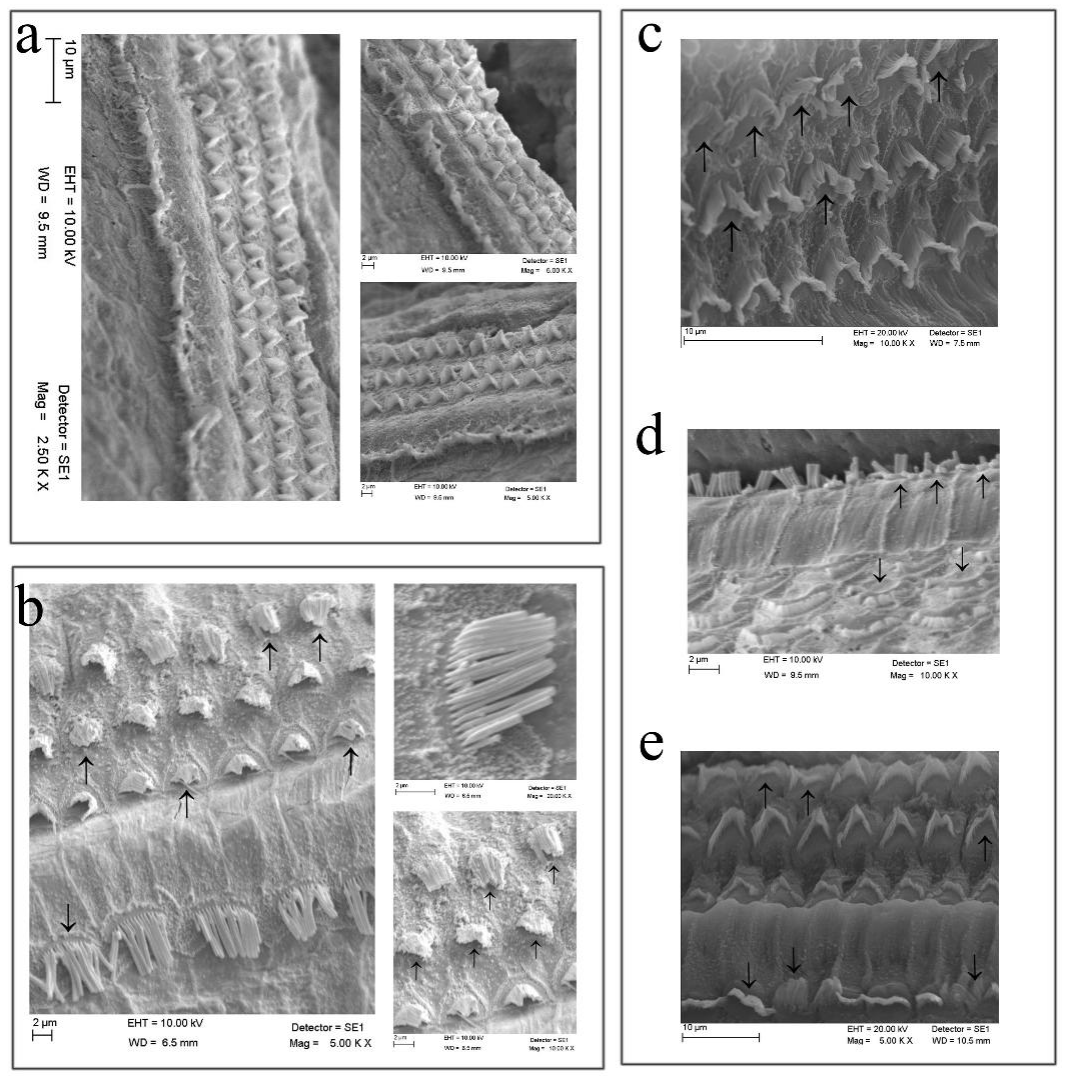
Morphological changes (→) of the cochlea in the groups. a) IHCs and OHCs stereocilia were observed as having little degeneration in the sham group b) HCs stereocilia partial and total losses were observed in all turns of cochlea in the PE group c). In the PEM group, IHCs stereocilia was observed as having less degeneration in all turns of cochlea, and OHCs stereocilia partial loss was observed in all turn of cochlea d) In the PEV group, IHCs and OHCs stereocilia were observed as having less degeneration and rupture in all turns of cochlea e) In the PEMV group, IHCs and OHCs stereocilia were observed to have less degeneration in all turn of cochlea.

In the sham group, IHC and OHC stereocilia showed less degeneration in all turns of the cochlea (Figure 4a). In the PE group, IHC and OHC stereocilia had degeneration and rupture in all turns of the cochlea. Partial HC stereocilia and total losses were observed in all turns of the cochlea. (Figure 4b). In the PEM group, IHC stereocilia showed less degeneration in all turns of the cochlea. Partial loss of OHC stereocilia was observed in all turns of the cochlea (Figure 4c). In the PEV group, IHC and OHC stereocilia had less degeneration and rupture in all turns of the cochlea, and partial HC stereocilia loss was observed in all turns of the cochlea. (Figure 4d). In the PEMV group, IHC and OHC stereocilia showed less degeneration in all turns of the cochlea (Figure 4e)

## 4. Discussion

The current study aimed to investigate the preventive/therapeutic effects of Vit D, MgSO_4_, and Vit D + MgSO_4_ supplementation on cochlear damage in a rat model of PE. In doing so, we used a preeclamptic rat model obtained using RUPP [10]. Our results indicate that the systolic BP of PE rats significantly increased compared to the sham group, demonstrating that the RUPP model was successfully implemented.

Several studies have indicated that the supplementation of Vit D or MgSO_4_ has a beneficial effect on PE-related pathology. In a study investigating the potential therapeutic effects of Vit D supplementation in a rat model of PE, Vit D2 and Vit D3 were given to the normal pregnant rats and PE rats during 14–18 days of pregnancy, and arterial BPs were then measured [11]. In addition to a significant decrease in arterial BP of PE rats on the 19th day of gestation, a significant decrease was detected in the levels of angiotensin II type 1 autoantibody, endothelin-1 secreted from the renal cortex, and plasma-dissolved FMS-like tyrosine kinase-1. However, several studies have shown a negative relationship between 25-hydroxyvitamin D, a circulating form of Vit D, and blood pressure [12], plasma 25-hydroxyvitamin D, and essential hypertension. Based on these findings, it was suggested that Vit D supplementation may play a role in the prevention or treatment of hypertensive diseases of pregnancy in response to placental ischemia [11]. In another study, Palacios et al. (2016) reported that oral Vit D supplementation during pregnancy could suppress the development of PE [13]. Consistent with the studies mentioned above, we also show that Vit D supplementation at 14.5 and 19.5 dpc has beneficial effects on maternal BP and fetal outcomes, such as EW and ES, in PE rats. Vit D supplementation enabled BP to return to normal values and induced a statistically significant increase in EW in the PEV group compared to PE. However, Vit D supplementation was insufficient in preventing the decrement of ES induced by PE.

Although the action mechanism of Mg^2+^ in the pathophysiology of PE is highly controversial because of conflicting findings in the literature, the primary reason for these contradictory results is the heterogeneity or differences in the sample size. In addition, a recent study demonstrated that the serum Mg^2+^ levels in PE and normal pregnant rats are significantly lower than in nonpregnant rats [14]. These findings increase the complexity of determining whether a deficiency of Mg^2+^ plays an important role in the development of PE. However, in another study, researchers investigated the protective effect of MgSO_4_ on pathological conditions originating from PE. In doing so, they noted changes in arterial BP, the protein level in cerebrospinal fluid (CSF), albumin concentration, and cytokine levels in PE and normal pregnant rats. The findings show that MgSO_4_ administration did not affect arterial BP and fetal outcomes in normal pregnant rats, but it reduced cerebral edema and significantly decreased the albumin and cytokine levels of CSF in the PE rats [15]. Consistent with these findings, we observed that MgSO_4_ supplementation at 14.5 and 19.5 dpc significantly benefited maternal BP by preventing increased arterial BP induced by PE and fetal outcomes by increasing EW. However, the ES of the PEM group remained lower than the sham group’s ES, which was also observed in the PEV group. Based on our results, it might be concluded that in the case of a pathology caused by PE, which can cause embryo death, the supplementation of Vit D and MgSO_4_ separately does not affect ES, even if it ameliorated the EW of live pups. In addition, when used together, the effects of Vit D and MgSO_4_ led to more effective results than when used separately. We observed that administering Vit D + MgSO_4_ had statistically significant beneficial effects on maternal BP, EW, and ES in the PEMV group. Therefore, a combined administration of Vit D and MgSO4 during pregnancy may be more beneficial than using them separately against the pathology related to PE.

Increasing evidence has indicated that hearing loss is an important complication associated with PE. Some PE-related complications, such as hearing loss, may be permanent even if PE is resolved/treated after delivery. Some researchers have reported that PE leads to failure of cochlear OHC function; however, no exact information about the reason for this dysfunction exists [2,16]. In this study, we showed that PE leads to destruction in the cochlear morphology. Degeneration and rupture of all turns of the cochlea in the PE group indicate that PE may lead to permanent destruction in the cochlea; this damage also explains why pregnant women with PE experience permanent hearing loss even after delivery. Cochlear degeneration is believed to result from increased oxidative stress in the PE group, as many studies have shown that PE is strongly associated with oxidative stress—although we did not measure the level of oxidative stress in the present study [17,18]. Furthermore, when evaluating the possible protective effects of combined or separate administration of Vit D and MgSO_4_ on cochlear degeneration in the PE group, we observed that the supplementation of Vit D and/or MgSO_4_ at levels ranging from 14.5 and 19.5 dpc resulted in important improvements in PE-related cochlear degeneration. Considering the antioxidant effect of Vit D and the neuroprotective effect of MgSO_4_, it is suggested that Vit D and/or MgSO_4_ administration during pregnancy can potentially prevent oxidative damage and related pathology in PE.

The main limitation of this study is that the cochlea was evaluated only morphologically to determine the pathology associated with PE. However, we propose that determining whether or not the morphological changes caused by PE have any functional effect should also be evaluated as electrophysiological. Therefore, extensive electrophysiological studies in which researchers can obtain recordings of electrocochleography and auditory-evoked potentials are required to discover the dysfunction observed in the auditory pathways related to PE.

In conclusion, we observed the increased arterial BP and decrement of the ES and EW in a PE group to demonstrate that the RUPP model is an appropriate animal model for studying PE-related pathologies. In addition, our results indicate that Vit D and MgSO4 have beneficial effects on PE pathology; administering Vit D and/or MgSO_4_ during pregnancy may also play an essential role in preventing PE-related complications, including cochlear degeneration.
